# Exploring intrinsically disordered proteins using site-directed spin labeling electron paramagnetic resonance spectroscopy

**DOI:** 10.3389/fmolb.2015.00021

**Published:** 2015-05-19

**Authors:** Nolwenn Le Breton, Marlène Martinho, Elisabetta Mileo, Emilien Etienne, Guillaume Gerbaud, Bruno Guigliarelli, Valérie Belle

**Affiliations:** Bioénergétique et Ingénierie des Protéines Laboratory, UMR 7281, Aix-Marseille Université and Centre National de la Recherche ScientifiqueMarseille, France

**Keywords:** intrinsically disordered proteins, structural transitions, induced folding, fuzzy complex, nitroxide spin labels, site-directed spin labeling EPR spectroscopy

## Abstract

Proteins are highly variable biological systems, not only in their structures but also in their dynamics. The most extreme example of dynamics is encountered within the family of Intrinsically Disordered Proteins (IDPs), which are proteins lacking a well-defined 3D structure under physiological conditions. Among the biophysical techniques well-suited to study such highly flexible proteins, Site-Directed Spin Labeling combined with EPR spectroscopy (SDSL-EPR) is one of the most powerful, being able to reveal, at the residue level, structural transitions such as folding events. SDSL-EPR is based on selective grafting of a paramagnetic label on the protein under study and is limited neither by the size nor by the complexity of the system. The objective of this mini-review is to describe the basic strategy of SDSL-EPR and to illustrate how it can be successfully applied to characterize the structural behavior of IDPs. Recent developments aimed at enlarging the panoply of SDSL-EPR approaches are presented in particular newly synthesized spin labels that allow the limitations of the classical ones to be overcome. The potentialities of these new spin labels will be demonstrated on different examples of IDPs.

## Introduction

Characterizing dynamics of macromolecules is a complex task requiring the use of appropriate techniques. In parallel to the development of methods leading to structure resolution of proteins, there is also an increasing need to develop biophysical techniques able to describe structural flexibility, as this dynamical aspect is closely related to protein function (Dyson and Wright, 2002; Salmon et al., 2010). The most highly dynamic biological systems are the so-called Intrinsically Disordered Proteins (IDPs) or Regions (IDRs), which lack a well-defined 3D structure under physiological conditions while being associated to key functions such as regulation, molecular assembly, signaling… (for recent reviews see Uversky and Dunker, 2010; Babu et al., 2011; Chouard, 2011; Habchi et al., 2014). Among the various techniques able to give access to dynamic properties of biomolecules is Site-Directed Spin Labeling combined with Electron Paramagnetic Resonance (SDSL-EPR), a technique that was pioneered about 20 years ago by (Hubbell et al., 1996). SDSL-EPR is very sensitive for probing flexible regions of proteins and revealing dynamic changes or interaction sites in protein-protein interactions (Belle et al., 2007; Lorenzi et al., 2012). It can also be used to determine accessibility profiles, a powerful approach to determine the topology of membrane proteins (Gross et al., 1999; Kaplan et al., 2000). More recently, the development of pulse EPR, in particular Double Electron Electron Resonance (DEER) techniques allowed the measurement of distances between spin labeled sites in the range of 1.8–6.0 nm (Pannier et al., 2000), thus covering a wide range of interest for the study of large conformational transitions and biomolecule associations. Excellent reviews describing all of these approaches, as well as applications of SDSL-EPR on proteins, have been recently published (Klare and Steinhoff, 2009; Bordignon, 2011; Mchaourab et al., 2011; Drescher, 2012; Hubbell et al., 2013). The present contribution will focus on dynamic analyses of extremely flexible biological systems and recent synthesis of new spin labels designed to enlarge the potentialities of the technique.

## General principles and development of new spin labels

**Figure 1:**
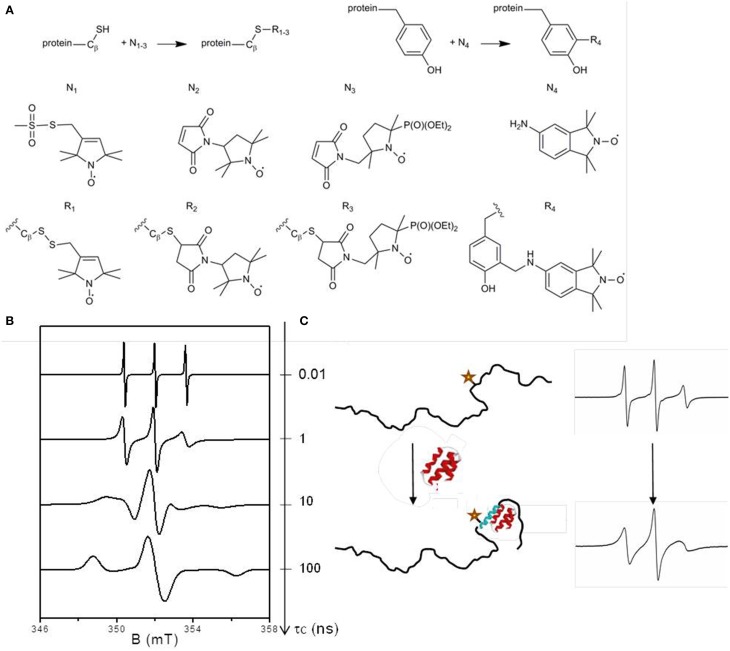
**(A)** Labeling reaction scheme for cysteine and tyrosine residues. N_1−3_ are spin labels for grafting on cysteine residues: N_1_ is the methanethiosulfonate spin label (MTSL), N_2_ the maleimido proxyl (P) and N_3_ the phosphorylated maleimido proxyl (PP). N_4_ is the an isoindoline-based nitroxide (Nox) dedicated to tyrosine labeling. R_1−4_ are the corresponding spin label side-chains. **(B)** EPR spectral shape modifications as a function of the mobility of the spin label described by its rotational correlation time τ_c_. The spectra were simulated using the EasySpin software (Stoll and Schweiger, 2006) for different values of τ_c_: 0.01, 1, 10, and 100 ns. **(C)** Illustration of an EPR spectral shape broadening in the case of a disorder-to-order transition due to an induced folding mechanism. The star represents the spin label.

SDSL-EPR relies on selective grafting of a paramagnetic species, usually a nitroxide derivative, at a desired position of a protein and subsequent analysis by EPR spectroscopy. Nitroxides are stable radicals in which the unpaired electron is delocalized on the N-O group, leading to a 3-line spectrum arising from the hyperfine interaction between the electron spin and the nuclear spin of the ^14^N atom. Nitroxide spin labels are classically functionalized to react specifically with cysteine residues. The technique thus requires the construction of cysteine mutants in order to target specific sites. The most frequently used nitroxide spin label is the MTSL (1-oxyl-2,2,5,5-tetramethylpyrroline-3-methyl) methanethiosulfonate leading to the formation of a disulphide bridge between the side-chain of the cysteine and the label (Figure 1A, side-chain R_1_). Its relatively small size minimizes the potential perturbation of the biological system. Other commercial spin labels are available, such as the maleimide-functionalized ones having the advantage of forming a thio-ether bond with the protein side-chain preventing the label release in the presence of reducing agents. One example of such spin label is the 3-maleimido-2,2,5,5-tetramethyl-pyrrolidinyloxy referred to as Proxyl or P depicted in Figure 1A (spin label N_2_). These labels are, however, more sterically demanding than MTSL, and they can react with amines at high pH (Hideg et al., 2005). As for all labeling techniques, control experiments are essential for comparing wild-type protein and labeled mutants to check the non-invasiveness of the label with respect to protein structural and functional properties. By investigating the relationship between the mobility of the MTSL nitroxide side-chain and the structural elements of the T4 lysozyme taken as a model protein, Mchaourab and co-workers established the basis for the interpretation of EPR spectral shapes of spin labels (Mchaourab et al., 1996). The power of the technique relies on the sensitivity of the EPR spectral shapes to the mobility of the label in the nanosecond time window described by the rotational correlation time τ_c_ (Figure 1B), a parameter that can be obtained by spectral simulation. Indeed the magnetic hyperfine interaction between the electron spin and the ^14^N nucleus is highly anisotropic. This anisotropy is fully averaged when the radical is highly mobile and the spectrum displays three narrow lines. When the mobility decreases, the averaging becomes partial and the lines broaden progressively until reaching a limit of a fully anisotropic spectrum corresponding to an immobilized spin label. A spectral modification thus represents a change in the environment of the label affecting its mobility and thus indicates a structural transition (Figure 1C).

The technique has still some limitations, in particular due to the chemical nature of the spin labels, which our recent works aim to overcome. One limitation comes from the poor diversity of EPR spectral shapes of nitroxide labels. The similarity of the 3-line spectral shapes precludes the simultaneous investigation of two different regions of a protein or two interacting proteins, a situation that can be encountered in allosteric mechanism in which ligand binding at one site influences binding at another site through a propagated structural change within the protein. To overcome this limitation, we designed and synthesized a new spin label based on a maleimido-functionalized β-phosphorylated nitroxide: the {2-(diethoxyphosphoryl)-5-[(2,5-dioxo-2,5-dihydro-1H-pyrrol-1-yl)methyl]-2,5 dimethylpyrrolidin-1-yl}oxidanyl, referred to as Phosphorylated Proxyl or PP (Figure 1A, N_3_). Thanks to the supplementary high magnetic coupling between the electron spin and the nuclear spin of ^31^P, this label gives a well-resolved 6-line spectrum composed of a doublet of triplets (Le Breton et al., 2014). Another limitation of conventional SDSL concerns the fact that only cysteines are specifically targeted by commercial nitroxides spin labels which is unsuitable when cysteines play roles either in the function and/or in structural elements (active sites, disulfide bridges) of the protein under study. We designed and synthesized various nitroxides to react with the phenol group of the tyrosine *via* a three-components Mannich type reaction (Lorenzi et al., 2011; Mileo et al., 2013a). The best results were obtained with an isoindoline-based nitroxide: 5-amino-1,1,3,3-tetramethyl-isoindolin-2-yloxyl, referred to as Nox (Figure 1A, N_4_), which has proved to be a good reporter of structural modifications (Mileo et al., 2013a). The successful applications of these two new nitroxides to report on structural transitions on IDPs will be presented in the next section.

## Applications

Several IDPs have already been investigated by SDSL-EPR. This approach has been successfully used to reveal various structural states and higher-order organizations of flexible proteins involved in neurodegenerative diseases such as α-synuclein (Chen et al., 2007), amyloid-β peptide (Torok et al., 2002) and tau (Siddiqua and Margittai, 2010). Another example of IDP studied by SDSL-EPR is the small acid protein IA3 that acts as an inhibitor of the yeast proteinase A (Casey et al., 2014). In the following, results with two highly flexible proteins are reviewed to illustrate the potential of SDSL-EPR in the field of IDPs.

### Cartography of induced folding

**Figure 2:**
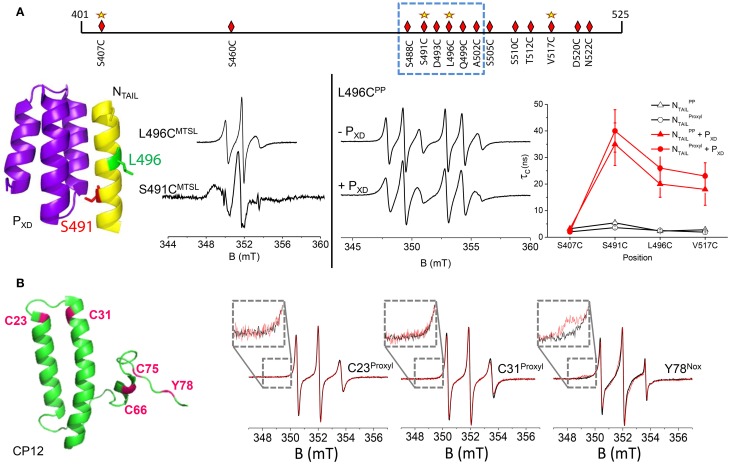
**(A)** upper panel: schematic representation of positions targeted for cysteine substitution and spin labeling of N_TAIL_ (aa 401–525) with MSTL spin label (diamonds) and PP spin label (stars). The dotted frame indicates the region that undergoes an induced folding in the presence of the partner protein P_XD_. Left panel: Illustration of two EPR spectral shapes obtained in two positions of the MTSL within the induced folding region of N_TAIL_: positions 491 and 496. These two positions are highlighted in the crystal structure of the chimera construct between P_XD_ and the N_TAIL_ region encompassing residues 486–504 (pdb code 1T60). Right panel: EPR spectra of the phosphorylated proxyl grafted at position 496 in the absence and in the presence of P_XD_. Variation of the rotational correlation time τ_c_ of Proxyl (circles) and Phosphorylated Proxyl PP (triangles) spin labeled N_TAIL_ variants without (black open symbols) and with (red filled symbols) saturating amounts of P_XD_ as a function of spin label position. τ_c_ values have been obtained by simulating the EPR spectra using the program ROKY (Rockenbauer and Korecz, 1996). **(B)** Left panel: 3D structural model of *C. reinhardtii* CP12 (pdb 2DDN) in which the positions of the four cysteines and the unique tyrosine are highlighted in pink. Right panel: Superimposition of amplitude-normalized EPR spectra of CP12 C23^Proxyl^, C31^Proxyl^, and Y78^Nox^ in absence (black) and presence of GAPDH in equimolar ratio (red). In inset: a zoom on the low-field region.

Most IDPs undergo an induced folding in presence of a partner protein i.e., a disorder-to-order transition that can be limited to a particular region. An illustrative example concerns the cartography of induced folding of nucleoproteins (N) from three viruses belonging to the *Paramyxoviridae* family, namely Measles (MeV), Nipah (NiV) and Hendra (HeV) viruses. Multiple copies of N are structurally organized to encapsidate the viral genome. The particular case of MeV nucleoprotein has been the most extensively studied by complementary biophysical approaches (Habchi and Longhi, 2012). MeV N consists of two regions: a N-terminal globular one (aa 1–400) and a C-terminal domain N_TAIL_ (aa 401–525) that is fully disordered. This disordered part is essential for transcription and replication of the virus *via* interaction with the phosphoprotein of the viral polymerase complex. Our study was focused on the interaction between MeV N_TAIL_ and the C-terminal part (X Domain) of the phosphoprotein P_XD_ (aa 459–507 of P), which is constituted of 3 α-helices (Longhi et al., 2003). In order to precisely localize the regional folding that MeV N_TAIL_ undergoes in the presence of P_XD_, we targeted 14 sites for spin-labeling (with MTSL) within N_TAIL_, 12 of which being concentrated in the C-terminal region (aa 488–525) that was known to be specifically involved in the interaction with MeV P_XD_ (Longhi et al., 2003) (Figure 2A, upper panel). For all labeled variants of N_TAIL_, room temperature EPR spectra were recorded in the absence and presence of P_XD_ (Belle et al., 2008). An important decrease of the mobility of the spin labels was observed in the 488–502 region that we have been able to attribute to the formation of an α-helix using complementary circular dichroism analyses. At one particular position (aa 491) a broad shape component was detected, indicating a very restricted environment of the spin label (Figure 2A, left panel). This observation allowed us to confirm the structural model of a chimera construct between MeV P_XD_ and a small N_TAIL_ region (aa 486–504) in which the amino acid side-chain 491 points toward P_XD_ whereas the other ones are solvent-exposed (Kingston et al., 2004). Interestingly, the mobility of the induced folding region was found to be slightly but significantly restrained even in the absence of the partner protein, a behavior that could indicate the existence of a pre-structuration of this region. This observation has been further confirmed combining SDSL-EPR data and modeling of local rotation conformational space (Kavalenka et al., 2010) and also by NMR studies and modeling of this region as a dynamic equilibrium between a completely unfolded state and different partially helical conformations (Jensen et al., 2011). Concerning the C-terminal end of N_TAIL_, EPR spectral shapes of the labels grafted in the 505–525 region showed a moderate decrease of mobility that has been attributed to a gain of rigidity arising from α-helical folding of the neighboring 488–502 region (Belle et al., 2008). This observation was consistent with further analyses where the 505–525 region was found to conserve a significant degree of freedom even in the bound form (Kavalenka et al., 2010). Using the same strategy based on multiple individual labeling sites, we also mapped the induced folding of N_TAIL_ in association with P_XD_ for HeV and NiV viruses (Martinho et al., 2013) and validated previously proposed structural models obtained by homology modeling (Habchi et al., 2011).

Thanks to the well-characterized MeV N_TAIL_-P_XD_ interaction by conventional spin labels, this biological system was used as a model to characterize the new label PP, having a ^31^P atom in the vicinity of the nitroxide leading to a 6-line spectrum (Figure 1A, N_3_) and to probe its ability to report structural transitions. Four grafting sites on N_TAIL_ were judiciously chosen (within and outside the induced folding region) (Figure 2A, upper panel). For comparative purposes, proxyl P (Figure 1A, side-chain R_2_) was grafted at the same positions. The ability of PP to report on structural transitions was evaluated by analyzing the spectral shape modifications induced either by a secondary structure stabilizer (trifluoroethanol) or by the presence of the partner protein P_XD_ (Figure 2A, right panel). All the spectra were simulated to extract the dynamic parameter τ_c_ that represents the mobility of the spin labels. The modification of this parameter according to the position of the spin label and the condition (free or bound to P_XD_) was very similar for both the classical proxyl and the new phosphorylated spin labels (Figure 2B, right panel). Taken together the results demonstrated that PP is able to monitor from subtle to larger structural transitions, as efficiently as the classical spin label. Molecular dynamics (MD) calculations were performed to gain further insights into the binding process between the labeled N_TAIL_ and P_XD_. MD calculations revealed that the new phosphorylated label does not perturb the interaction between the two partner proteins and reinforced the conclusion on its ability to probe different local environments in a protein (Le Breton et al., 2014). Thanks to its new EPR spectral signature, the combination of PP with classical spin labels opens the way to study two protein sites simultaneously.

### Revealing fuzziness in a supramolecular complex

In this section we will show how SDSL-EPR provides a unique tool to reveal regions of an IDP remaining highly flexible after complex formation, information that often escapes to detection by other biostructural techniques. This study concerns the structural flexibility of a small chloroplastic protein called CP12 (80 aa) and its association with the glyceraldehyde-3-phosphate dehydrogenase (GAPDH) from the green alga *Chlamydomonas reinhardtii*. This association is a key step in the formation of a ternary supramolecular complex involved in the regulation of the Calvin cycle in many photosynthetic organisms (for details see the review Tieulin-Pardo et al in the present journal). In its oxidized state the green alga CP12 contains four cysteine residues engaged in two disulfide bridges (C23–C31 and C66–C75) and presents some α-helical structural elements modeled from each side of the N-terminal disulfide bridge, whereas the C-terminal part appears mainly disordered (Figure 2B) (Gardebien et al., 2006). In contrast, in its reduced state the algal CP12 is fully disordered (Graciet et al., 2003). In a first study, we used MTSL (Figure 1A, N_1_), which led to the labeling of the only C-terminal cysteine residues leaving the N-terminal unmodified as revealed by mass spectrometry analyses. Surprisingly, the partner protein GAPDH induced the cleavage of the disulfide bridge between the cysteine and the label, resulting in the full release of the label. This result showed the existence of a transitory interaction between both proteins and we proposed a mechanism based on a thiol-disulfide exchange reaction involving cysteines C21 and C291 of the *C. reinhardtii* GAPDH (Erales et al., 2009). Even if this observation led us to propose a new role for the algal GAPDH, it however doesn't allow us to study the GAPDH-CP12 complex. As the central region of CP12 has been proposed to be involved in the interaction with GAPDH, we used two cysteine-to-serine mutants (C23S and C31S) that were known to be fully disordered, due to the loss of the N-terminal disulfide bridge, but still able to interact with GAPDH (Lebreton et al., 2006). In order to individually target the two N-terminal cysteine residues, proxyl P was chosen as spin label (Figure 1A, N_2_) to prevent label release induced by GAPDH. EPR analyses revealed that this part of the protein remains fully disordered after association with GAPDH as no spectral modification was detected (Figure 2B) (Mileo et al., 2013b). The association between the two partner proteins was checked by probing the accessibility of the labels using a reducing agent. Indeed, in the absence of GAPDH, spin labels grafted on CP12 are reduced by an excess of DTT and become EPR-silent with a characteristic time of about 25 min. On the contrary, if GAPDH is present in the sample, the same amount of DTT has almost no effect as the EPR signal remains stable for at least 2 h confirming that the association really takes place and that CP12 remains highly dynamic in its bound form (Mileo et al., 2013b).

The C-terminal region has been demonstrated to be mainly responsible for the redox regulation of GAPDH (Lebreton et al., 2006). To gain further insights into the dynamics of this region of CP12 while keeping the 2 disulfide bridges intact, tyrosine 78 was chosen as an alternative labeling site. The location of tyrosine 78, highly conserved in CP12s from different organisms, is particularly interesting as it is close to residues that play a crucial role in the activity modulation of GAPDH either in the binary complex (Erales et al., 2011) or in the ternary complex with PRK (Avilan et al., 2012). The new isoindoline-based nitroxide Nox (Figure 1A, N4) was used to selectively target this unique tyrosine. The presence of GAPDH led to a slight modification of the EPR spectral shape (Figure 2B) indicating that the interaction of the C-tail with the partner protein is not tight. This result showed however that this region is close to the interaction site without being directly involved in it (Mileo et al., 2013b). All together the analyses of the three labeling sites were in good agreement with the partial view of the CP12-GAPDH complexes from other organisms given by recent crystallographic and NMR studies where only the 20 last amino acids of CP12 were detected (Matsumura et al., 2011; Fermani et al., 2012). Finally, the use of different spin labels is this peculiar biological system allowed us to conclude that GAPDH-CP12 from *C. reinhardtii* is a new example of a fuzzy complex, a concept introduced by Tompa and Fuxreiter (2008), in which the IDP keeps most of its disorder and dynamics upon complex formation. This fuzziness could be one of the keys to facilitate the formation of a ternary complex (CP12, GAPDH and phosphoribulokinase, PRK) as well as functional actions of the machinery which necessitate dynamic assembly and disassembly processes controlled by dark/light transitions.

## Conclusion

The examples described above, along with numerous other studies, illustrate the power of SDSL-EPR to access information on protein dynamics. Characterized by a remarkable conformational flexibility, IDPs form a unique protein category that is particularly suited for SDSL-EPR applications. SDSL-EPR is a rapidly growing field, in particular with recent developments focused on the detection of labeled protein in intact cells, an area that promises to be very interesting for IDPs applications. We can anticipate that these recent developments will create further need for the design of new labels with greater stability toward bio-reduction, an environment that is encountered inside the cell.

### Conflict of interest statement

The authors declare that the research was conducted in the absence of any commercial or financial relationships that could be construed as a potential conflict of interest.
